# *N*-(4-Cyano­benz­yl)benzamide

**DOI:** 10.1107/S1600536808035137

**Published:** 2008-11-08

**Authors:** Yi-Li Tong, Li-Qin Guo, Hai-Jun Ma, Wei Chen, Hong-Jun Zhu

**Affiliations:** a Department of Applied Chemistry, College of Science, Nanjing University of Technology, Nanjing 210009, People’s Republic of China; b Jiangsu Pesticide Research Institute Co. Ltd, Nanjing 210009, People’s Republic of China

## Abstract

The title compound, C_15_H_12_N_2_O, is a derivative of 4-(amino­meth­yl)benzonitrile, an important pestcide inter­mediate. In the crystal structure, mol­ecules are linked *via* inter­molecular N—H⋯O hydrogen bonds, forming infinite chains.

## Related literature

For general background, see: Blaschke *et al.* (1976 ▶); Gesing (1989 ▶). For the synthetic procedure, see: Guo *et al.* (2008 ▶). For bond-length data, see: Allen *et al.* (1987 ▶).
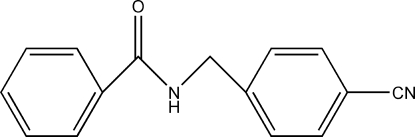


## Experimental

### 

#### Crystal data

C_15_H_12_N_2_O
*M* *_r_* = 236.27
Monoclinic, 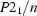

*a* = 5.864 (1) Å
*b* = 27.164 (5) Å
*c* = 7.839 (2) Å
β = 91.09 (3)°
*V* = 1248.4 (4) Å^3^
*Z* = 4
Mo *K*α radiation
μ = 0.08 mm^−1^
*T* = 298 (2) K
0.30 × 0.20 × 0.10 mm

#### Data collection

Enraf–Nonius CAD-4 diffractometer
Absorption correction: ψ scan (North *et al.*, 1968 ▶) *T*_min_ = 0.976, *T*_max_ = 0.992
2450 measured reflections
2233 independent reflections
1461 reflections with *I* > 2σ(*I*)
*R*_int_ = 0.041
3 standard reflections every 200 reflections intensity decay: none

#### Refinement

*R*[*F* ^2^ > 2σ(*F* ^2^)] = 0.075
*wR*(*F* ^2^) = 0.186
*S* = 1.00
2233 reflections
163 parameters
H-atom parameters constrained
Δρ_max_ = 0.26 e Å^−3^
Δρ_min_ = −0.24 e Å^−3^

### 

Data collection: *CAD-4 Software* (Enraf–Nonius, 1985 ▶); cell refinement: *CAD-4 Software*; data reduction: *XCAD4* (Harms & Wocadlo, 1995 ▶); program(s) used to solve structure: *SHELXS97* (Sheldrick, 2008 ▶); program(s) used to refine structure: *SHELXL97* (Sheldrick, 2008 ▶); molecular graphics: *SHELXTL* (Sheldrick, 2008 ▶); software used to prepare material for publication: *SHELXTL*.

## Supplementary Material

Crystal structure: contains datablocks I, global. DOI: 10.1107/S1600536808035137/im2085sup1.cif

Structure factors: contains datablocks I. DOI: 10.1107/S1600536808035137/im2085Isup2.hkl

Additional supplementary materials:  crystallographic information; 3D view; checkCIF report

## Figures and Tables

**Table 1:** Hydrogen-bond geometry (Å, °)

*D*—H⋯*A*	*D*—H	H⋯*A*	*D*⋯*A*	*D*—H⋯*A*
N2—H2*A*⋯O^i^	0.86	1.99	2.830 (4)	166

Symmetry code: (i) 
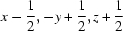
.

**Fig. 1.**
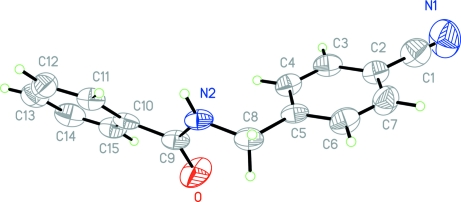
Molecular structure of the title compound showing the atom-numbering scheme. Displacement ellipsoids are drawn at the 50% probability level.

**Fig. 2.**
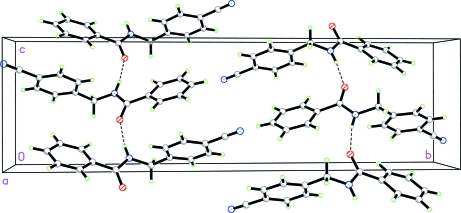
Packing diagram of (I). Hydrogen bonds are shown as dashed lines.

## supplementary crystallographic information

### Comment

*N*-(4-Cyanobenzyl)benzamide is a derivative of 4-(aminomethyl)benzontrile (Gesing, 1989), which is an important in the synthesis of pestcides as well as of some drugs (Blaschke *et al.*, 1976).

The molecular structure of (I) is shown in Fig. 1. The bond lengths and angles are within normal ranges (Allen *et al.*, 1987).

In the crystal structure, molecules are linked together to form infinite chains *via* intermolecular N—H···O hydrogen bonds (Fig. 2).

### Experimental

The title compound, (I) was prepared by a method reported by Guo *et al.* (2008).

Crystals were obtained by dissolving (I) (0.8 g, 3.4 mmol) in dichloromethane (20 ml) and slowly evaporating the solvent slowly at room temperature for about 5 d.

### Refinement

H atoms were positioned geometrically, with N—H = 0.86 and C—H = 0.93Å for aromatic H, and constrained to ride on their parent atoms, with *U*_iso_(H) = *xU*_eq_(C/N), where *x* = 1.2 for aromatic H and *x* = 1.5 for other H.

### Crystal data

**Table:**

C_15_H_12_N_2_O	*F*_000_ = 496
*M**_r_* = 236.27	*D*_x_ = 1.257 Mg m^−^^3^
Monoclinic, *P*2_1_/*n*	Mo *K*α radiation λ = 0.71073 Å
Hall symbol: -P 2yn	Cell parameters from 25 reflections
*a* = 5.864 (1) Å	θ = 10–13º
*b* = 27.164 (5) Å	µ = 0.08 mm^−^^1^
*c* = 7.839 (2) Å	*T* = 298 (2) K
β = 91.09 (3)º	Block, colorless
*V* = 1248.4 (4) Å^3^	0.30 × 0.20 × 0.10 mm
*Z* = 4	

### Data collection

**Table:**

Enraf–Nonius CAD-4 diffractometer	*R*_int_ = 0.041
Radiation source: fine-focus sealed tube	θ_max_ = 25.3º
Monochromator: graphite	θ_min_ = 1.5º
*T* = 298(2) K	*h* = −7→7
ω/2θ scans	*k* = 0→32
Absorption correction: ψ scan(North *et al.*, 1968)	*l* = 0→9
*T*_min_ = 0.976, *T*_max_ = 0.992	3 standard reflections
2450 measured reflections	every 200 reflections
2233 independent reflections	intensity decay: none
1461 reflections with *I* > 2σ(*I*)	

### Refinement

**Table:**

Refinement on *F*^2^	Secondary atom site location: difference Fourier map
Least-squares matrix: full	Hydrogen site location: inferred from neighbouring sites
*R*[*F*^2^ > 2σ(*F*^2^)] = 0.075	H-atom parameters constrained
*wR*(*F*^2^) = 0.186	*w* = 1/[σ^2^(*F*_o_^2^) + (0.06*P*)^2^ + 2*P*] where *P* = (*F*_o_^2^ + 2*F*_c_^2^)/3
*S* = 1.00	(Δ/σ)_max_ < 0.001
2233 reflections	Δρ_max_ = 0.26 e Å^−^^3^
163 parameters	Δρ_min_ = −0.24 e Å^−^^3^
Primary atom site location: structure-invariant direct methods	Extinction correction: none

### Special details

**Table:**

Geometry. All e.s.d.'s (except the e.s.d. in the dihedral angle between two l.s. planes) are estimated using the full covariance matrix. The cell e.s.d.'s are taken into account individually in the estimation of e.s.d.'s in distances, angles and torsion angles; correlations between e.s.d.'s in cell parameters are only used when they are defined by crystal symmetry. An approximate (isotropic) treatment of cell e.s.d.'s is used for estimating e.s.d.'s involving l.s. planes.
Refinement. Refinement of *F*^2^ against ALL reflections. The weighted *R*-factor *wR* and goodness of fit *S* are based on *F*^2^, conventional *R*-factors *R* are based on *F*, with *F* set to zero for negative *F*^2^. The threshold expression of *F*^2^ > σ(*F*^2^) is used only for calculating *R*-factors(gt) *etc*. and is not relevant to the choice of reflections for refinement. *R*-factors based on *F*^2^ are statistically about twice as large as those based on *F*, and *R*- factors based on ALL data will be even larger.

### Fractional atomic coordinates and isotropic or equivalent isotropic displacement parameters (Å^2^)

**Table:**

	*x*	*y*	*z*	*U*_iso_*/*U*_eq_	
O	0.3309 (5)	0.24546 (11)	0.3711 (3)	0.0728 (9)	
C1	0.5980 (7)	0.02472 (18)	0.7639 (6)	0.0648 (11)	
N1	0.7125 (8)	−0.00565 (17)	0.8071 (6)	0.0976 (15)	
N2	0.0883 (4)	0.23194 (10)	0.5795 (3)	0.0396 (7)	
H2A	0.0269	0.2426	0.6711	0.048*	
C2	0.4555 (6)	0.06509 (14)	0.7054 (4)	0.0465 (9)	
C3	0.5228 (6)	0.11328 (14)	0.7289 (4)	0.0473 (9)	
H3A	0.6602	0.1201	0.7852	0.057*	
C4	0.3894 (5)	0.15128 (13)	0.6701 (4)	0.0422 (8)	
H4A	0.4383	0.1836	0.6850	0.051*	
C5	0.1830 (5)	0.14198 (12)	0.5889 (4)	0.0354 (8)	
C6	0.1169 (6)	0.09393 (14)	0.5685 (5)	0.0514 (9)	
H6A	−0.0224	0.0873	0.5146	0.062*	
C7	0.2468 (7)	0.05557 (15)	0.6239 (5)	0.0571 (10)	
H7A	0.1971	0.0234	0.6077	0.069*	
C8	0.0315 (5)	0.18301 (13)	0.5198 (4)	0.0431 (8)	
H8A	0.0384	0.1828	0.3963	0.052*	
H8B	−0.1247	0.1759	0.5501	0.052*	
C9	0.2321 (5)	0.26091 (13)	0.4974 (4)	0.0385 (8)	
C10	0.2683 (5)	0.31165 (12)	0.5607 (3)	0.0332 (7)	
C11	0.1042 (5)	0.33738 (13)	0.6512 (4)	0.0407 (8)	
H11A	−0.0302	0.3217	0.6809	0.049*	
C12	0.1384 (6)	0.38530 (14)	0.6968 (5)	0.0528 (10)	
H12A	0.0297	0.4020	0.7596	0.063*	
C13	0.3361 (7)	0.40892 (15)	0.6489 (5)	0.0550 (10)	
H13A	0.3575	0.4420	0.6745	0.066*	
C14	0.5014 (6)	0.38324 (16)	0.5631 (5)	0.0542 (10)	
H14A	0.6378	0.3986	0.5362	0.065*	
C15	0.4657 (5)	0.33607 (14)	0.5182 (4)	0.0447 (9)	
H15A	0.5765	0.3195	0.4572	0.054*	

### Atomic displacement parameters (Å^2^)

**Table:**

	*U*^11^	*U*^22^	*U*^33^	*U*^12^	*U*^13^	*U*^23^
O	0.094 (2)	0.0745 (19)	0.0522 (15)	−0.0141 (16)	0.0520 (15)	−0.0124 (14)
C1	0.052 (3)	0.075 (3)	0.067 (3)	0.006 (2)	−0.011 (2)	−0.005 (2)
N1	0.087 (3)	0.079 (3)	0.125 (4)	0.026 (2)	−0.026 (3)	0.003 (3)
N2	0.0334 (15)	0.0591 (18)	0.0267 (13)	0.0019 (13)	0.0090 (11)	−0.0020 (12)
C2	0.044 (2)	0.055 (2)	0.0412 (19)	0.0021 (17)	0.0006 (16)	−0.0035 (16)
C3	0.0340 (19)	0.067 (2)	0.0407 (19)	−0.0052 (17)	−0.0050 (15)	−0.0033 (17)
C4	0.0356 (18)	0.053 (2)	0.0385 (18)	−0.0108 (16)	0.0028 (14)	−0.0059 (15)
C5	0.0255 (16)	0.055 (2)	0.0256 (15)	0.0002 (14)	0.0042 (12)	−0.0041 (13)
C6	0.038 (2)	0.063 (2)	0.053 (2)	−0.0110 (18)	−0.0145 (17)	−0.0085 (18)
C7	0.056 (2)	0.050 (2)	0.064 (2)	−0.0027 (19)	−0.013 (2)	−0.0099 (19)
C8	0.0338 (18)	0.060 (2)	0.0356 (17)	−0.0021 (16)	−0.0016 (14)	0.0004 (15)
C9	0.0300 (17)	0.061 (2)	0.0250 (15)	0.0000 (15)	0.0120 (13)	0.0029 (14)
C10	0.0218 (15)	0.057 (2)	0.0206 (14)	0.0046 (14)	−0.0001 (12)	0.0082 (13)
C11	0.0242 (16)	0.061 (2)	0.0365 (17)	0.0016 (15)	0.0004 (13)	0.0025 (15)
C12	0.052 (2)	0.057 (2)	0.049 (2)	0.0076 (19)	−0.0028 (17)	−0.0064 (18)
C13	0.057 (2)	0.058 (2)	0.049 (2)	−0.011 (2)	−0.0165 (19)	0.0015 (18)
C14	0.037 (2)	0.076 (3)	0.049 (2)	−0.0154 (19)	−0.0079 (17)	0.0077 (19)
C15	0.0305 (18)	0.070 (3)	0.0343 (17)	0.0034 (17)	0.0040 (14)	0.0054 (16)

### Geometric parameters (Å, °)

**Table:**

O—C9	1.230 (4)	C7—H7A	0.9300
C1—N1	1.113 (5)	C8—H8A	0.9700
C1—C2	1.448 (6)	C8—H8B	0.9700
N2—C9	1.329 (4)	C9—C10	1.479 (5)
N2—C8	1.446 (4)	C10—C15	1.380 (4)
N2—H2A	0.8600	C10—C11	1.394 (4)
C2—C3	1.379 (5)	C11—C12	1.364 (5)
C2—C7	1.394 (5)	C11—H11A	0.9300
C3—C4	1.370 (5)	C12—C13	1.383 (5)
C3—H3A	0.9300	C12—H12A	0.9300
C4—C5	1.380 (4)	C13—C14	1.380 (5)
C4—H4A	0.9300	C13—H13A	0.9300
C5—C6	1.370 (5)	C14—C15	1.344 (5)
C5—C8	1.519 (5)	C14—H14A	0.9300
C6—C7	1.357 (5)	C15—H15A	0.9300
C6—H6A	0.9300		
			
N1—C1—C2	178.1 (5)	N2—C8—H8B	108.4
C9—N2—C8	122.1 (3)	C5—C8—H8B	108.4
C9—N2—H2A	118.9	H8A—C8—H8B	107.5
C8—N2—H2A	118.9	O—C9—N2	120.0 (3)
C3—C2—C7	118.9 (3)	O—C9—C10	121.4 (3)
C3—C2—C1	121.0 (3)	N2—C9—C10	118.5 (3)
C7—C2—C1	120.1 (3)	C15—C10—C11	118.2 (3)
C4—C3—C2	120.7 (3)	C15—C10—C9	118.9 (3)
C4—C3—H3A	119.7	C11—C10—C9	122.8 (3)
C2—C3—H3A	119.7	C12—C11—C10	120.8 (3)
C3—C4—C5	120.5 (3)	C12—C11—H11A	119.6
C3—C4—H4A	119.8	C10—C11—H11A	119.6
C5—C4—H4A	119.8	C11—C12—C13	119.4 (4)
C6—C5—C4	118.2 (3)	C11—C12—H12A	120.3
C6—C5—C8	119.7 (3)	C13—C12—H12A	120.3
C4—C5—C8	122.1 (3)	C14—C13—C12	119.9 (4)
C7—C6—C5	122.6 (3)	C14—C13—H13A	120.1
C7—C6—H6A	118.7	C12—C13—H13A	120.1
C5—C6—H6A	118.7	C15—C14—C13	120.1 (3)
C6—C7—C2	119.1 (4)	C15—C14—H14A	119.9
C6—C7—H7A	120.4	C13—C14—H14A	119.9
C2—C7—H7A	120.4	C14—C15—C10	121.5 (3)
N2—C8—C5	115.5 (3)	C14—C15—H15A	119.3
N2—C8—H8A	108.4	C10—C15—H15A	119.3
C5—C8—H8A	108.4		
			
C7—C2—C3—C4	−1.3 (5)	C8—N2—C9—C10	175.9 (3)
C1—C2—C3—C4	178.7 (3)	O—C9—C10—C15	−22.3 (5)
C2—C3—C4—C5	1.2 (5)	N2—C9—C10—C15	158.1 (3)
C3—C4—C5—C6	−0.3 (5)	O—C9—C10—C11	152.8 (3)
C3—C4—C5—C8	−179.2 (3)	N2—C9—C10—C11	−26.8 (4)
C4—C5—C6—C7	−0.4 (5)	C15—C10—C11—C12	−0.2 (5)
C8—C5—C6—C7	178.5 (3)	C9—C10—C11—C12	−175.4 (3)
C5—C6—C7—C2	0.2 (6)	C10—C11—C12—C13	1.6 (5)
C3—C2—C7—C6	0.7 (6)	C11—C12—C13—C14	−3.2 (5)
C1—C2—C7—C6	−179.3 (4)	C12—C13—C14—C15	3.4 (5)
C9—N2—C8—C5	90.5 (4)	C13—C14—C15—C10	−2.1 (5)
C6—C5—C8—N2	166.8 (3)	C11—C10—C15—C14	0.4 (5)
C4—C5—C8—N2	−14.4 (4)	C9—C10—C15—C14	175.8 (3)
C8—N2—C9—O	−3.7 (5)		

### Hydrogen-bond geometry (Å, °)

**Table:**

*D*—H···*A*	*D*—H	H···*A*	*D*···*A*	*D*—H···*A*
N2—H2A···O^i^	0.86	1.99	2.830 (4)	166

Symmetry codes: (i) *x*−1/2, −*y*+1/2, *z*+1/2.
